# Food handler's level of COVID-19 prevention practice and preparedness of food and drinking establishments to combat the pandemic in Eastern Ethiopia

**DOI:** 10.3389/fpubh.2022.912077

**Published:** 2022-08-15

**Authors:** Sisay Habte, Adera Debella, Tilahun Abdeta, Abdi Birhanu, Bikila Balis, Bajrond Eshetu, Habtamu Bekele

**Affiliations:** ^1^School of Nursing and Midwifery, College of Health and Medical Sciences, Haramaya University, Harar, Ethiopia; ^2^School of Medicine, College of Health and Medical Sciences, Haramaya University, Harar, Ethiopia

**Keywords:** coronavirus, preventive, practice, preparedness, food, drinking, establishment, Ethiopia

## Abstract

**Background:**

Food and drinking establishments are potential hotspots for the spread of coronavirus. Food handler's have a higher risk of contracting the disease and transmitting it to others. The aim of this study was to assess the food handler's level of preventive practices toward COVID-19 and the preparedness of food and drinking establishments to tackle the pandemic in Eastern Ethiopia.

**Methods:**

The cross-sectional study design was conducted from September 1 to 30, 2020. A stratified sampling technique was used to select 276 licensed public food and drinking establishments and a simple random sampling technique was employed to select 422 food handlers from the selected establishments. A face-to-face interview and checklist-based observation were used to collect data. The collected data were entered into Epidata 3.1 and exported to STATA version 16 for analysis. Binary logistic regression analysis were conducted to identify factors associated with COVID-19 preventive practice. Statistical significance was declared at *p* < 0.05.

**Results:**

About 406 food handler's participated in this study, making the response rate 96.2%. The study showed that 38.4% of study participants (95% CI: 33.5, 43.1) had good preventive practices for COVID-19. Only 10.5% of food and drinking establishments fulfilled all requirements to prevent COVID-19 transmission. Being male [AOR = 0.61, 95% CI(0.61, (0.39, 0.93)], attending secondary education [AOR = 2.20, (95% CI: 1.37, 3.53)], having a favorable attitude toward COVID-19 [AOR = 1.89, (95% CI: 1.22, 2.95)], and having good knowledge about COVID-19 [AOR = 1.78, (95% CI: 1.13, 2.81)] were significantly associated with the level of COVID-19 preventive practices.

**Conclusion:**

The level of good COVID-19 preventive practice was found to be low among the food handler's. Only one in ten food and drink establishments fulfilled the national guideline for preventing COVID-19 transmission. Being male, attending secondary education, having knowledge about COVID-19, and having a favorable attitude toward COVID-19 were significantly associated with good COVID-19 preventive practices. A vibrant guideline on prevention practices should be in place at all establishments, and compliance should be monitored. Local health office experts should take comprehensive measures to make all food and drinking establishments accountable for practicing all preventive measures.

## Introduction

Coronavirus disease (COVID-19) is an infectious disease caused by the novel coronavirus 2 (SARS-CoV-2) (1). On 17 November 2019, the first confirmed COVID-19 case was reported in Wuhan, China, and then the disease quickly spread across the world (1–3). In January 2020, it was declared as a public health emergency of international concern, and it became declared a pandemic in March 2020 (4). In Africa, the first case of COVID-19 was reported in Egypt on 14 February 2020, and Ethiopia reported the first confirmed COVID-19 case on 13 March 2020 (2, 5). The disease causes morbidity extending from mild respiratory illness to severe acute respiratory distress syndrome, metabolic, and homeostasis disorders, and finally leads to death (6).

Thousands of people died of COVID-19, and the disease aggravated the current social, political, and socioeconomic problems in the entire population on the globe (7, 8). Furthermore, COVID-19 has contributed to the discontinuation of academic activities. However, e-learning systems became an optional tool to sustain education during the COVID-19 pandemic (9).

Initially, COVID-19 was considered zoonotic (animal-to-human). However, it is mainly transmitted human-to-human through direct and indirect contact (10, 11). Primarily, COVID-19 spreads through respiratory droplets produced by infected patients while talking, coughing, or sneezing (12). Moreover, it is also spread *via* indirect contact, such as when a person touches his or her eyes, nose, or mouth with contaminated hands (13). All asymptomatic, symptomatic, and recovered patients can transmit the virus (14, 15). COVID-19 chiefly affects the respiratory system, but it also involves other organ systems (16). The symptoms range from mild to severe (17). Dry cough, fever, shortness of breath, myalgia, headache, disorientation, sore throat, hemoptysis, runny nose, chills, chest pain, and rhinorrhea are the most common symptoms associated with COVID-19. It also leads to admission to an intensive care unit and a high mortality rate (16, 18).

The prevention of COVID-19 transmission requires adequate awareness and individual willingness to adhere to preventive practices (19, 20). Surface disinfection; frequent hand washing; physical distancing; avoiding touching the mouth, nose, and eyes; and wearing personal protective equipment (PPE) are recommended preventive measures for coronavirus infection (21, 22). A systematic review from Iran indicated the use of N95 respirators and adherence to the principles of personal hygiene, frequent hand washing, and the use of disinfectants can reduce the prevalence of COVID-19 (23).

Even though COVID-19 is not foodborne, improper food handling can lead to infection, and food handlers can spread the virus (24–26). Evidence from previous studies showed that plastic surfaces can serve as vectors for COVID-19 transmission as the virus can last for several hours and days on plastic surfaces. In addition, equipment like plastics (e.g., single-use plastics, plastic bags, plastic bottles, and styrofoam containers for food packing) used in food and drinking establishments can be potential physical vectors or fomites of SARS-CoV-2, and this indicates the need to decontaminate and disinfect plastic surfaces used or associated with food and drinks (27–29).

It is known that many individuals visit food and drinking establishments (FDE) from different areas. As a result, food handlers might get infected and transmit this disease to others (30). Consequently, WHO strongly recommended that individuals especially those who work in crowded areas like food and drinking establishments should practice appropriate COVID-19 preventive practices (25, 31). Several studies indicated that factors, such as availability of supplies, knowledge about COVID-19, attitude toward COVID-19, and socio-demographic characteristics of individuals, can affect their practice toward COVID-19 (32, 33). In addition, the insight self-efficacy, perceived severity of the COVID-19, and intention to meet the needs of preventive measures along with misconceptions and unconfirmed beliefs in the general public also contribute for preventive practice toward COVID-19 prevention (34).

Harar and Dire Dawa are two of the most at-risk areas as they are near Djibouti, which is the one of the gate to the country. Thus, travelers and residents share similar food and drinking establishments, which may exacerbate the chance of cross-infection. Therefore, the preparedness of FDE to reduce the chance of cross-contamination is very important (35). Even though some studies were conducted on the practice of individuals at the community level, there is no study conducted assessing food and drinking establishments' preparedness toward to combat COVID-19 and the practice of food handlers working in the establishment. Therefore, the aim of this study was to assess the level of preventive practice for COVID-19 among food handlers in food and drinking establishments and the preparedness of the establishments to tackle the pandemic in Eastern Ethiopia.

## Methods and materials

### Study design, period, and setting

A cross-sectional study design was conducted from 1 to 30 September 2020 at Harar and Dire Dawa towns. Harar is located in the eastern part of Ethiopia, which is 526 km away from the capital city, Addis Ababa. In Harar, there were 282 licensed food and drinking establishments, including 69 hotels, 95 bars and restaurants, 89 cafes, and 29 groceries. Similarly, Dire Dawa is located 515 km east of the capital city, Addis Ababa. There were 446 licensed food and drinking establishments in Dire Dawa, including 81 hotels, 155 bars and restaurants, 110 cafes, and 100 groceries. In those licensed food and drinking establishments, there were 1,090 and 1,697 food handlers in Harar and Dire Dawa, respectively.

### Population

All licensed food and drinking establishments, as well as all food handlers working in these establishments in Harar and Dire Dawa, were the source population for this study. The study included food handlers above the age of 18 years. This study excluded food and drinking establishments that served as quarantine centers. Food handlers on any type of leave were excluded from this study.

### Sample size determination

#### Sample size determination for food and drinking establishments

The sample size for food and drinking establishments was calculated using a single population proportion formula with a 95% confidence interval, a 5% margin of error (d), and *p* = 50%. The calculated sample size was 384. Because the total number of food and drinking establishments in both towns was <10,000, the population correction formula was used. Therefore, the final sample size, including a 10% non-response rate, was 276.

#### Sample size determination for food handlers

Similarly, the sample size for food handlers was calculated using a single population proportion formula with a 95% confidence interval, a 5% margin of error (d), and *p* = 50%. The calculated sample size was 384, and when a 10% non-response rate was added, the final sample size was 422.

### Sampling procedures

#### Sampling procedure to select food and drinking establishments

**Figure 1:**
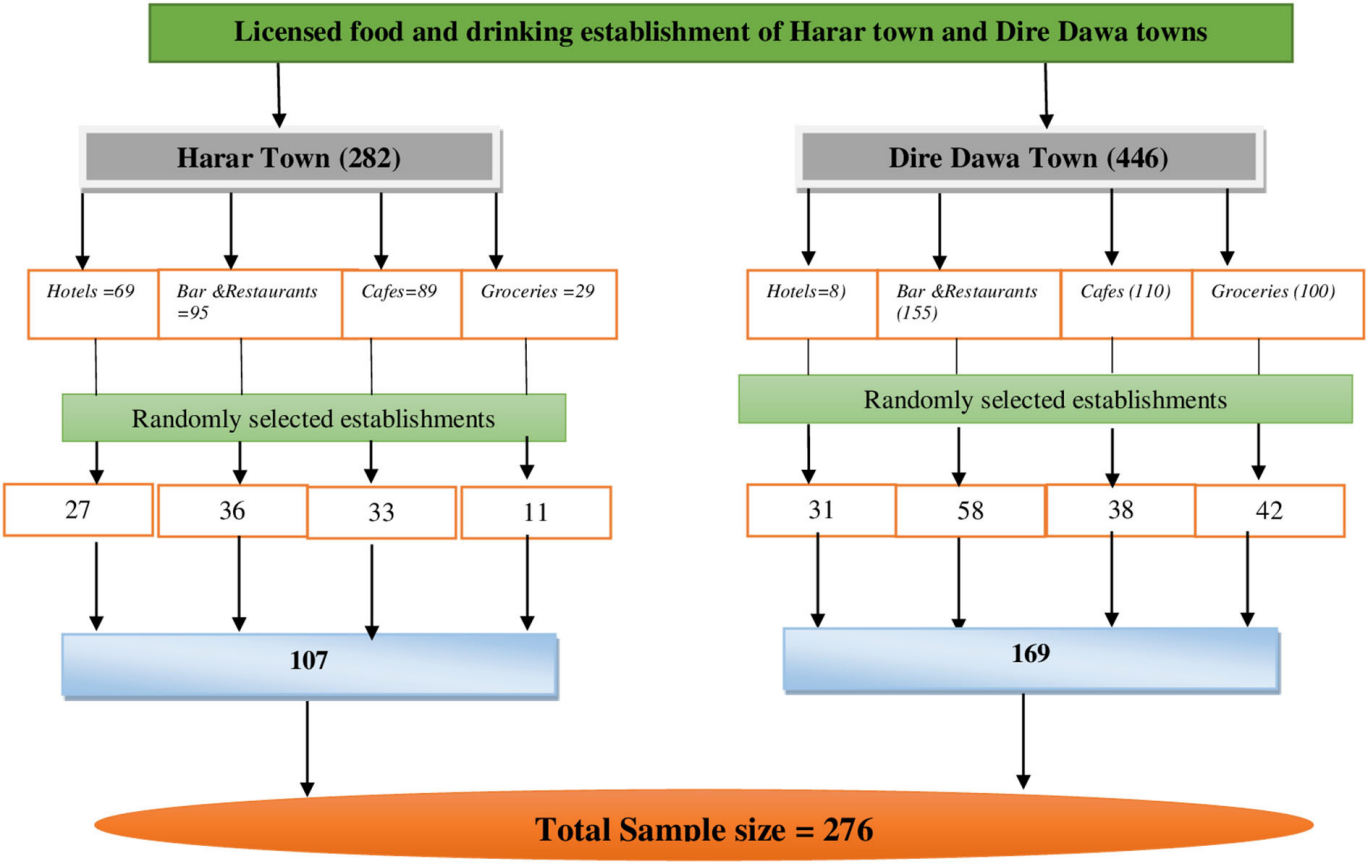
Schematic diagram of the sampling procedure for licensed food and drinking establishments in Harar and Dire Dawa, 2020.

A stratified sampling technique was utilized to select food and drinking establishments at Harar and Dire Dawa. After the establishments were stratified based on their location (Harar and Dire Dawa) and type (hotels, café, bar and restaurants, and groceries), the required sample of 276 was selected at random (Figure 1).

#### Sampling procedure to select food handlers

**Figure 2:**
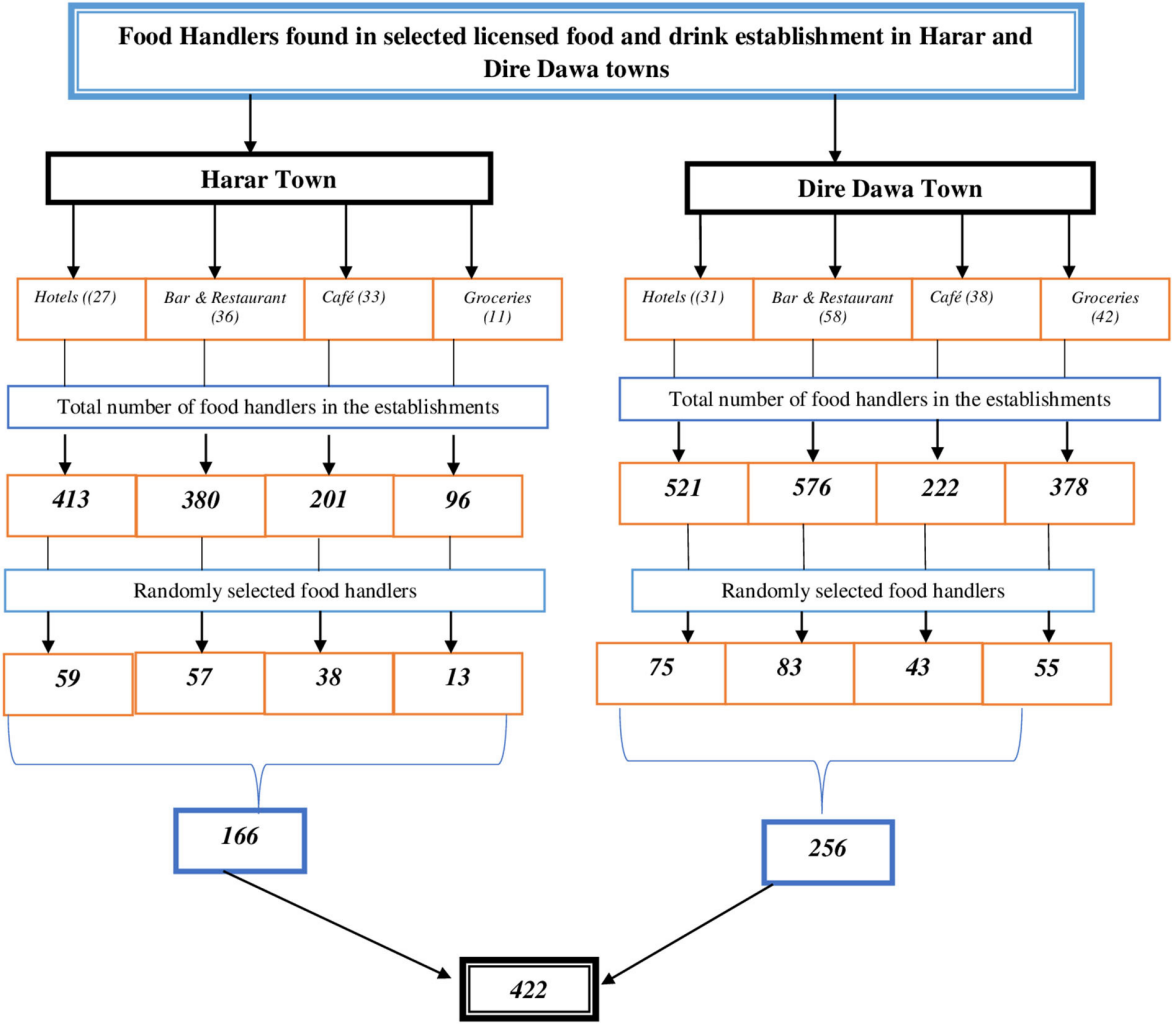
Schematic diagram of the sampling procedure of food handlers at selected licensed food and drinking establishments in Harar and Dire Dawa, 2020.

After proportional allocation of samples to each of the selected 276 licensed food and drinking establishments, a simple random sampling procedure was employed to select 422 food handlers (Figure 2).

### Measurement

Knowledge of COVID-19 (transmission, signs and symptoms, and prevention) was measured using 30 questions with three responses (no, not sure, and yes). The response was dichotomized. Participants who answered the correct answer were given one, while wrong responses were given 0. Participants who scored ≥80% on the total knowledge question were considered as having good knowledge of COVID-19.

Attitude toward COVID-19 was measured by 12 items on a 5-point Likert scale ranging from strongly agree to strongly disagree. The response was dichotomized. The correct answer, which represented a positive attitude, was computed as one, and the erroneous answer, which represented a negative attitude, was computed as 0. Participants who scored ≥80% on the total attitude questions were considered to have a favorable attitude (36, 37).

The level of preventive practice of COVID-19 was measured by 10 questions with three responses (no, not sure, and yes). Then, the response was dichotomized into good and poor practices. Participants who gave the correct response were given 1 point, while those who gave incorrect answers were given 0 point. The score ranges from 0 to 10. Participants who scored ≥80% on the total practice-related questions were considered to have good preventive practice (38).

The preparedness of the establishments to prevent COVID-19 transmission was measured using an observational checklist prepared based on the national guidelines. The checklist was mainly comprised of physical distancing requirements, the availability of a functional handwashing facility, and staff face mask utilization. The establishments that met all of these requirements were considered prepared.

### Data collection procedures

Data were collected through face to face interview using a structured questionnaire and an observational checklist, which were developed after reviewing various studies (39–41). The questionnaries contains sociodemographic characteristics and COVID-19-related information. The questionnaire were initially prepared in English and then translated into Amharic and Afan Oromo and then back into English by language experts to check its consistency. Data were collected by five health professionals and supervised by the principal investigator.

### Data quality control

To ensure the quality of the data, a structured and pretested questionnaire was used. A pretest was done with 5% of the total sample size at Haramaya town. The questionnaires were modified based on the pretest results, repetitive ideas and ambiguous questions were corrected, and the modified questionnaires were used for the final data collection. For two days, data collectors were trained on data collection methods and interview techniques. Proper categorization and coding of questionnaires were critically prepared before the data collection. Furthermore, the principal investigator was meticulously checking the collected data on a daily basis for completeness, accuracy, and clarity.

### Data processing and analysis

After data collection, the questionnaire was checked for its completeness, cleaned, and coded. The collected data were entered into Epidata 3.1 and exported to STATA version 16 for further analysis. Simple frequencies and percentages were used to summarize data. A binary logistic regression analysis was performed to check factors associated with preventive practice toward COVID-19 among food handlers. Based on findings, variables with a *p* < 025 in the bivariate logistic regression analysis were considered in the multivariable logistic regression analysis. A multivariable adjusted odds ratio (AOR) along with a 95% confidence interval was estimated to assess the strength of the association, and a *p* < 0.05 was used to declare the statistical significance in the multivariable analysis. Hosmer and Lemeshow's goodness-of-fit was used to assess whether the necessary assumptions were fulfilled. The model was considered a good fit since it was found to be insignificant for the Hosmer–Lemeshow statistic (*p* = 0.341). Finally, the results were presented *via* text, tables, and figures.

## Result

### Sociodemographic characteristics of study participants

The survey received responses from 406 food handlers, with a response rate of 96.2%. Males made up more than half of the participants in this study 210 (51.7%). The mean age of study participants was 27.28 (SD = 8.1). The majority of the participants in the study 192 (47.3%) worked in hotels. Almost half 202 (49.8%) of the participants attended at least primary education. The majority of the study participants, 382 (94.1%), were urban dwellers. Moreover, the majority, 384 (94.6%), had ≤ 5 years of work experience (Table 1).

**Table 1:** Sociodemographic characteristics of food handlers at food and drinking establishments at Harar and Dire Dawa, 2020 (*N* = 406).

**Variables**	**Categories**	**Frequency**	**Percentage**
Sex	Female	196	48.3
	Male	210	51.7
Age in year	<25	276	68.0
	26–35	96	23.6
	>35	34	8.4
Marital status	Never married	258	63.5
	Ever married	148	36.5
Religion	Muslim	115	28.3
	Orthodox	235	57.9
	Protestant	50	12.3
	Others	6	1.5
Educational status	Primary	202	49.8
	Secondary	144	35.5
	Tertiary	60	14.8
Working establishment	Hotel	192	47.3
	Bar and restaurant	77	19.0
	Café	56	13.8
	Grocery	81	20.0
Residence	Urban	382	94.1
	Rural	24	5.9
Working experience in year	≤ 5	384	94.6
	≥6	22	5.4

### Knowledge of food handler's on COVID-19

**Figure 3:**
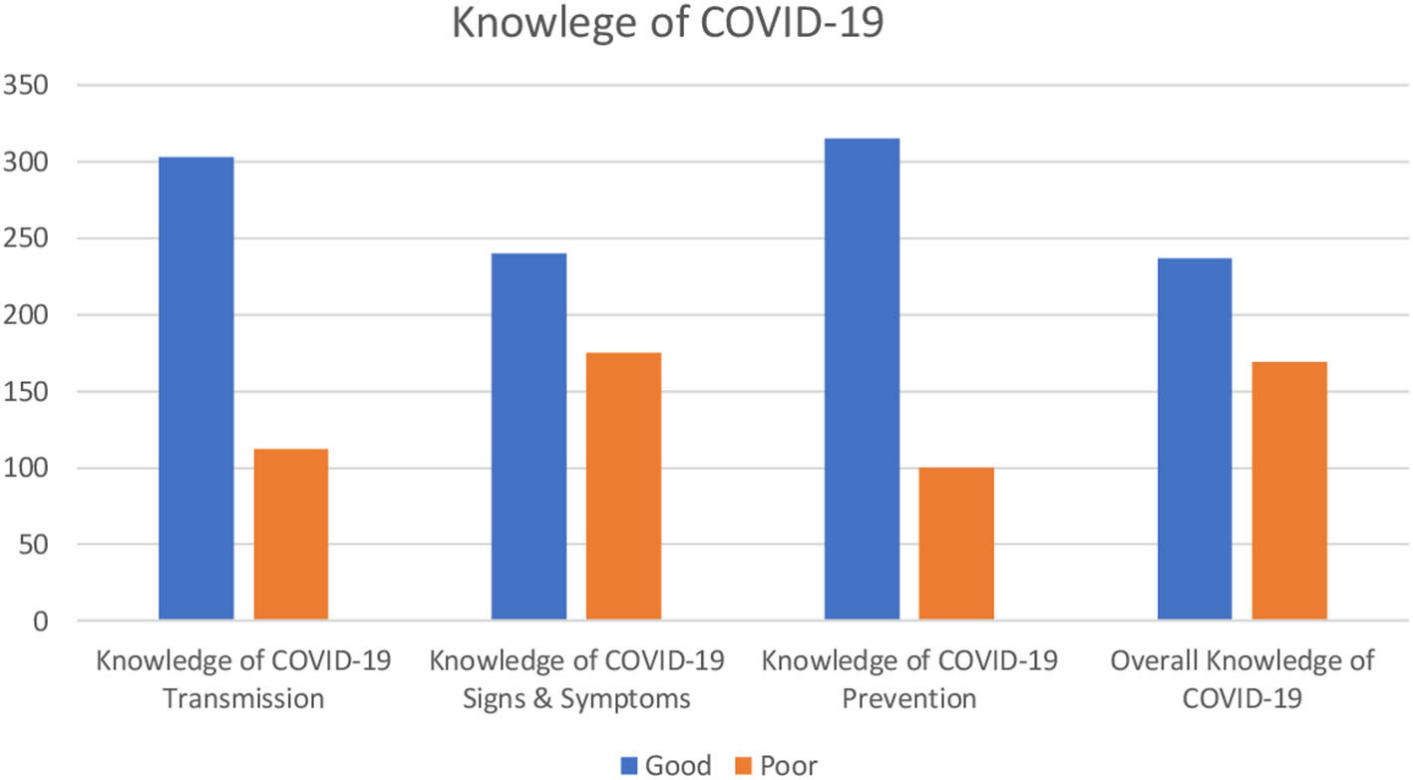
Knowledge of food handlers at food and drinking establishments on COVID-19 in Harar and Dire Dawa, 2020.

The overall proportion of food handler's with good knowledge about COVID-19 was 58.4% (95% CI: 53.9, 63.1). About 73, 57.5, and 75.9% of study participants had good knowledge of COVID-19 transmission, signs and symptoms, and prevention, respectively (Figure 3). The majority of the participants, 391 (96.3%), responded that COVID-19 can transmit between people, whereas 96.3% responded that COVID-19 can transmit through respiratory droplets (Tables 2A–2C).

**Table 2A:** Knowledge of food handler's at food and drinking establishments toward COVID-19 transmission at Harar and Dire Dawa, 2020 (*N* = 406).

**S/N**	**Questions**	**Yes**	**No**
1.	Did the COVID-19 transmit from people to people?	399 (98.3%)	7 (1.7%)
2.	COVID-19 can spread through respiratory droplets produced when an infected person coughs, sneezes, or talks	391 (96.3%)	15 (3.7%)
3.	COVID-19 can spread by touching a surface or object that has the virus on it and then touching the mouth, nose, or eyes	391 (96.3%)	15 (3.7%)
4.	COVID-19 may spread between people who are in close contact with one another (usually <6 feet)	371 (91.5%)	35 (8.6%)
5.	COVID-19 may spread by eating raw meat and uncooked vegetables	308 (75.8%)	98 (24.1%)
6.	COVID-19 may be spread by people who are not showing symptoms	192 (47.3%)	124 (30.5%)
7.	COVID-19 can spread from animals to people in some situations	124 (30.5%)	282 (69.4%)
8.	COVID-19 can spread only through exported good	198 (46.51%)	222 (53.5%)
9.	COVID-19 can be transmitted through contact with the stool of an infected person (feco-oral route)	205 (50.5%)	201(49.5%)
10.	COVID-19 may be transmitted through the air (airborne)	280 (69%)	126 (31.1%)

**Table 2B:** Knowledge of food handler's at food and drinking establishments about signs and symptoms of COVID-19 at Harar and Dire Dawa, 2020 (*N* = 406).

**S/N**	**Questions**	**Yes**	**No**
1.	Fever	392 (96.6%)	14 (3.5%)
2.	Dry cough	390 (96.1%)	16 (3.9%)
3.	Backache	295 (72.6%)	141 (27.3%)
4.	Difficulty in breathing	351 (88.5%)	42 (10.3%)
5.	Sore throat	318 (78.3%)	88 (21.7%)
6.	Headache	359 (88.6%)	46 (11.3%)
7.	Fatigue	317 (78.1%)	89 (21.9%)
8.	Loss of appetite	286 (70.4%)	120 (29.5%)
9.	Diarrhea	174 (42.9%)	232 (57.2%)
10.	Loss of sense of smell and test	277 (68.2%)	129 (31.8%)

**Table 2C:** Knowledge of food handler's at food and drinking establishments about signs and symptoms of COVID-19 at Harar and Dire Dawa, 2020 (*N* = 406).

**S/N**	**Questions**	**Yes**	**No**
1.	Maintain a physical distance of at least 1 m (3 feet) between you and others	390 (96.1%)	16 (3.9%)
2.	Regularly and thoroughly clean your hands with an alcohol-based hand rub or wash them with soap and water	393 (96.8%)	13 (3.2%)
3.	Avoid going to crowded places	361 (88.9%)	45 (11%)
4.	Avoid touching eyes, nose, and mouth	344 (84.7%)	62 (15%)
5.	Cover your mouth and nose with your bent elbow or tissue when you cough or sneeze	362 (89.2%)	44 (8.9%)
6.	Cover your mouth and nose with a mask when going into crowded places	355 (87.4%)	51 (12.6%)
7.	Stay home and self-isolate even with minor symptoms such as cough, headache, or mild fever, until you recover	294 (72.4%)	112 (27.4%)
8.	Stay aware of the latest information on the COVID-19 outbreak from trusted sources	276 (66.7%)	135 (33.3%)
9.	Eating garlic and taking lemon juice prevent COVID-19 infection	162 (39.9%)	244 (60.1%)
10.	Staying home is the best way to prevent COVID-19	121 (29.8%)	285 (70.2%)

### Attitude of food handler's toward COVID-19

More than half of the study participants, 51.5 (95% CI: 46.8, 56.4), had a favorable attitude toward COVID-19. The majority of the study participants, 238 (58.6%), strongly believed that COVID-19 is a serious disease (Table 3).

**Table 3:** Attitude of food handler's at food and drinking establishments toward COVID-19 at Harar and Dire Dawa, 2020 (*N* = 406).

**S/N**	**Questions**	**Strongly agree**	**Agree**	**Neutral**	**Disagree**	**Strongly disagree**
1.	COVID-19 is a serious disease	238 (58.6%)	124 (30.5%)	19 (4.7%)	17 (4.2%)	8(2%)
2.	You have the chance of getting customers infected with COVID-19 in your establishment	184 (45.3%)	164 (40.4%)	32 (7.9%)	20 (4.9%)	6(1.5%)
3.	In my opinion, early detection of COVID-19 can improve treatment and outcome	169 (41.6%)	153 (37.7%)	46 (11.3%)	30 (7.4%)	8(2.0%)
4.	In my opinion, COVID-19 can be treated at home	111 (27.3%)	171 (42.1%)	54 (13.3%)	54 (13.3%)	16 (3.9%)
5.	In my opinion, COVID-19 is not a curable disease that results in death in all cases	83 (20.4)	104 (25.6%)	64 (15.8%)	101 (24.9%)	54 (13.3%)
6.	As far as washing our hands no need to stay at home	94 (23.2%)	125 (30.5%)	79 (19.5%)	71 (17.5%)	37 (9.1%)
7.	It is my opinion that COVID-19 disease can be transmitted through household pets to humans	108 (26.6%)	139 (34.2%)	71 (17.3%)	51 (12.6%)	37 (9.1%)
8.	It is my opinion that authorities should restrict travel to and from COVID-19 disease areas to prevent contamination	91 (22.4%)	106 (26.1%)	79 (19.5%)	70 (17.2%)	60 (14.8%)
9.	Since COVID-19 is a foreign disease and as I have no travel history, I am safe	81 (20%)	95 (23%)	77 (19%)	91 (23.4%)	62 (15.3%)
10.	I feel safe when I interact with people by using a face mask	108 (26.6%)	165 (40.6%)	80 (19.7%)	38 (9.4%)	15 (3.7%)
11.	Death from COVID-19 is rare and I am not very much worried about it.	90 (22.2%)	135 (33.3%)	99 (24.4%)	53 (13.1%)	29 (7.1%)
12.	I believe there are some vulnerable individuals for COVID-19 than the general public	139 (34.2%)	152 (37.4%)	61 (15%)	37 (9.1%)	17 (4.2%)
13.	There is no worry about getting the disease because of eating raw meat	55 (13.5%)	70 (17.2%)	109 (26.8%)	80 (19.7%)	92 (22.7%)

### Food handler's level of preventive practice on COVID-19

The proportion of good preventive practice on COVID-19 among study participants was 38.4 (95% CI: 33.8, 43.1). The majority of the participants, 351 (86.5%), reported that they were washing their hands frequently. About 279 (68.7%) and 361 (88.9%) of the study participants avoided going to crowded places and shaking hands with people, respectively (Table 4).

**Table 4:** Preventive practice of food handlers at food and drinking establishments toward COVID-19 at Harar and Dire Dawa, 2020 (*N* = 406).

**S/N**	**Question**	**Yes**	**No**
1.	Do you regularly and thoroughly clean your hands with an alcohol-based hand rub or wash them with soap and water?	351 (86.5%)	55 (13.5%)
2.	Do you maintain a physical distance of at least 2 m (6 feet) between you and others at your work and other places?	310 (76.4%)	95 (23.6 %|)
3.	Do you avoid touching eyes, nose, and mouth with unwashed hands?	298 (73.4%)	108 (26.6%)
4.	Do you avoid going to crowded places?	279 (68.7%)	127 (31.3%)
5.	Do you stay at home unless for work and necessary activities?	273 (67.2%)	133 (82.8)
6.	Do you wear a mask when out in public places and at your workplace in recent days?	231 (56.9%)	175 (43.1%)
7.	Do you avoid touching the front of your mask frequently or during removing it?	279 (68.7%)	127 (31.2%)
8.	Do you cover your mouth and nose with your bent elbow or tissue when you cough or sneeze all the time?	218 (53.2%)	188 (46.8%)
9.	Do you avoid direct contact with people like handshaking and kissing?	361 (88.9%)	45 (11%)
10.	Are you taking balanced nutrition to keep yourself healthy	190 (46.8%)	216 (46.3%)

### Preparedness of food and drinking establishments to tackle COVID-19

A total of 266 foods and drinking establishments were involved in this study, making the response rate of 96.4%. The majority, 90 (33.8%), of these establishments included in this study were bars and restaurants. Cafes, groceries, and hotels account for 68 (25.6%), 51 (19.2%), and 57 (21.4%), respectively (Table 5). Only 28 (10.5%) of food and drinking establishments fulfilled all national requirements related to COVID-19 prevention. Approximately 195 (73.3%) food and drinking establishments had a handwashing station set up at their gate for customers (Table 6).

**Table 5:** Types of food and drinking establishments in Harar and Dire Dawa, 2020 (*N* = 266).

**S/N**	**Type of establishments**	**Frequencies**	**Percentages**
1.	Bar and restaurants	90	33.8
2.	Cafes	68	25.6
3.	Groceries	51	19.2
4.	Hotels	57	21.4
5.	Total	266	100.0

**Table 6:** Food and drinking establishments preparedness to tackle COVID-19 at Harar and Dire Dawa, 2020 (*N* = 266).

**S/N**	**Observational questions**	**Yes**	**No**
1.	Are the number of dining room tables arranged to accommodate only four customers within 10 m^2^?	141 (53%)	125 (47%)
2.	Is the distance between tables ≥2 m?	190 (71.4%)	76 (28.6)
3.	Is the distance between chairs ≥1 m?	188 (70.7%)	78 (29.3)
4.	Are staff working in the establishment maintaining physical distancing?	165 (62%)	101 (38)
5.	Is a functional handwashing facility arranged at the entrance for customers?	195 (73.3%)	71 (16.7)
6.	Is water available at the handwashing facility?	215 (80.8%)	51 (19.2%)
7.	Is soap available at the handwashing facility?	211 (79.3%)	55 (20.7%)
8.	Is hand sanitizer made available for all staff?	136 (51.1%)	130 (48.9%)
9.	Is cleaning chairs and tables with disinfectants done after every service?	128 (48.1%)	138 (51.9%)
10.	Is cleaning toilet door handles with disinfectants done frequently?	97 (36.5%)	169 (63.5%)
11.	Is cleaning of utensils with soap and water then by bleach done after every service?	147 (55.3%)	119 (44.7%)
12.	Do all staff put on masks?	131 (49.2%)	135 (50.8%)
13.	Does staff involve in cooking/preparing food put on gloves and masks while cooking/preparing?	80 (30.1%)	186 (69.9%)

### Factors associated with the level of preventive practice toward COVID-19 among food handler's

The relationship between each independent variable and the outcome variable was separately analyzed. Variables with a *p* < 0.25 were retained for the final multivariable logistic regression analysis. Variables like sex, educational status, knowledge, and attitude were significantly associated with the level of good COVID-19 preventive practice.

Male participants were 0.61 (AOR: 0.61, 95% CI: 0.39–0.93) times less likely to have good preventive practices than female participants. Participants who attended secondary and tertiary education were 2.20 (AOR: 2.20, 95% CI: 1.37–3.53) and 2.67 (AOR: 2.67, 95% CI: 1.44–4.96) times more likely to have good preventive practices than participants who attended primary education. The odds of having good preventive practices were 1.89 (AOR: 1.89, 95% CI: 1.22–2.95) times higher in participants with a favorable attitude toward COVID-19 than in their counterparts. Moreover, participants having good knowledge of COVID-19 were 1.78 (AOR: 1.78, 95% CI: 1.13–2.81) times more likely to have good preventive practices of COVID-19 than participants with poor knowledge of COVID-19 (Table 7).

**Table 7:** Factors associated with the level of preventive practice toward COVID-19 among food handlers at Dire Dawa and Harar, 2020.

**Variables**	**Level of practice**		**COR (95% CI)**	**AOR (95% CI)**	***P*-value**
	**Poor**	**Good**			
	**(*N* = 250)**	**(*N* = 156)**			
**Sex**				
Female	106	90	1	1	
Male	144	66	0.54 (0.36, 0.81)	**0.61 (0.39, 0.93)**	0.02
**Age in year**				
<25	172	104	1	1	
26–35	56	40	1.18 (0.73, 1.89)	0.99 (0.54, 1.81)	0.99
>35	22	12	0.90 (0.43, 1.89)	0.94 (0.38, 2.36)	0.90
**Marital status**				
Never married	164	94	1	1	
Ever married	86	62	1.26 (0.83, 1.90)	1.26 (0.73, 2.17)	0.40
**Educational status**				
Primary	145	57	1	1	
Secondary	77	67	2.21 (1.41, 3.46)	**2.20 (1.37, 3.53)**	0.001
Tertiary	28	32	2.90 (1.60, 5.26)	**2.67 (1.44, 4.96)**	0.001
**Working establishment**				
Hotel	123	69	1	1	
Bar and restaurant	45	32	1.26 (0.74, 2.18)	1.11 (0.61, 1.99)	0.72
Café	32	24	1.34 (0.73, 2.45)	1.30 (0.67, 2.51)	0.43
Grocery	50	31	1.11 (0.65, 1.89)	1.54 (0.85, 2.78)	0.15
**Residence**				
Urban	234	148	1	1	
Rural	16	8	0.79 (0.33, 1.89)	1.28 (0.49, 3.34)	0.60
**Working experience in years**				
≤ 5	237	147	1	1	
≥6	13	9	1.12 (0.46, 2.68)	0.96 (0.36, 2.52)	0.93
**Attitude on COVID-19**				
Unfavorable	136	61	1	1	
Favorable	114	95	1.86 (1.24, 2.79)	**1.89 (1.22, 2.95)**	0.001
**Knowledge on COVID-19**				
Poor	117	52	1	1	
Good	133	104	1.76 (1.16, 2.66)	**1.78 (1.13, 2.81)**	0.01

## Discussion

The aim of this study was to assess the level of preventive practice among food handler's working in the selected licensed public food and drinking establishments in Harar and Dire Dawa, Eastern Ethiopia, and the preparedness of these establishments to tackle the pandemic.

Accordingly, good preventive practices toword COVID-19 among food handler's in this study was 38.4 (95% CI: 33.8, 43.1). The finding of this study is almost consistent with the finding of a study conducted in Dessie City and Kombolcha Town, Ethiopia (43.9%) (42). However, this finding was higher than a study conducted in Southwest Ethiopia (21.2%) (37). This discrepancy might be due to the difference in study participants and study settings. This study was conducted mainly in an urban setting among workers in food and drinking facilities that entail food preparation, storage, or service.

In this study, male participants are less likely than female participants to have good COVID-19 preventive practices. The findings of the study were supported by studies conducted in Southwest Ethiopia (37), North-East Ethiopia (43), and Saudi Arabia (44). This could be explained by the fact that women are more cautious of themselves and others around them than men, which leads to better adherence to preventive measures (45).

This study pointed out that food handlers with a secondary and tertiary level of education were more likely to have good COVID-19 preventive practices as compared to those with primary level of education. This finding was consistent with studies conducted in Southwest Ethiopia (37), Dessie City and Kombolcha Town, Ethiopia (42), Uganda (46), and Iran (47). This could be explained by the fact that those with a higher level of education may have easier access to and comprehension of COVID-19 material. As a result, improved preventive behavior may be exhibited.

This study also showed that food handlers with good knowledge of COVID-19 were more likely to have a good level of COVID-19 preventive practice. This finding is supported by studies conducted in the Amhara Region, Ethiopia (32), Bangladesh (48), and Vietnam (49). This could be explained by the fact that if a person has sufficient knowledge of the disease's cause, transmission, and prevention, they are more likely to practice the prevention measures on purpose.

Moreover, the findings of this study revealed that food handlers with favorable attitudes toward COVID-19 were more likely to have good COVID-19 preventive practices. The findings from this study were supported by studies conducted in Nigeria (50), Bangladesh (51), and India (52). This is probably because positive outlooks consequently lead to adherence to prevention measures for COVID-19. This means if an individual feels positive toward the prevention of the disease, the more likely they will practice prevention methods.

In summary, this study revealed that only 38 (10.5%) of food and drinking establishments fulfilled all national requirements related to COVID-19 prevention. This finding is low and alarming as it was recommended that all food and drinking establishments be expected to adhere to the national and international precautions to be implemented for tackling the pandemic (31).

### Limitation of the study

Due to the nature of the study design, which is a cross-sectional study, the temporal relationship between the outcome and the exposure could not be established. In addition, the questionnaire was prone to social desirability bias. However, the study tried to address the study participants who were at higher risk of being infected with COVID-19 with an adequate sample size. Furthermore, this study was the first to address food and drinking establishments as a corner of the study suite for COVID-19.

## Conclusion

Only three out of five study participants had good knowledge of COVID-19, and half of them had a favorable attitude toward COVID-19. The level of good COVID-19 preventive practice was found to be low among the food handler's. In addition, only one out of ten food and drink establishments in Harar and Dire Dawa fulfilled the national guidelines for preventing COVID-19 transmission. Being male, attending secondary education, having good knowledge about COVID-19, and having a favorable attitude toward COVID-19 were significantly associated with good COVID-19 preventive practice. More information and assistance should be provided to encourage proper COVID-19 prevention behaviors. Establishment owners should monitor compliance with COVID-19 prevention behaviors and guide food handlers as needed. Local health office experts should take comprehensive measures to make all food and drinking establishments accountable for practicing all preventive measures.

## Data availability statement

The original contributions presented in the study are included in the article/supplementary material, further inquiries can be directed to the corresponding author.

## Ethics statement

Ethical approval was taken from the Institutional Health Research Ethics Review Committee (IHRERC) of the College of Health and Medical Sciences of Haramaya University. A letter of support was written to the selected food and drinking establishments. Informed, voluntary, written, and signed consent was obtained from all study participants. Confidentiality was assured throughout the study.

## Author contributions

SH was a principal investigator who initiated the research and participated on conception, study design, formal analysis, and writing original draft of the manuscript. HB, AD, TA, AB, BE, and BB participated in reviewing the analysis and writing the manuscript. All authors read and approved the final manuscript and accountable for all aspects of the study.

## Funding

Haramaya University has provided financial support for this study. The authors declared that the funding body has no role in designing the study, data collection, data analysis, and writing the manuscript.

## Conflict of interest

The authors declare that the research was conducted in the absence of any commercial or financial relationships that could be construed as a potential conflict of interest.

## Publisher's note

All claims expressed in this article are solely those of the authors and do not necessarily represent those of their affiliated organizations, or those of the publisher, the editors and the reviewers. Any product that may be evaluated in this article, or claim that may be made by its manufacturer, is not guaranteed or endorsed by the publisher.

## Abbreviations

AOR: adjusted odds ratio
CI: confidence interval
COR: crude odds ratio
FDE: food and drinking establishments
PPE: personal protective equipment
SD: standard deviation
WHO: World Health Organization.
